# Transarterial embolisation in the treatment of persistent haematuria in two dogs with lower urinary tract carcinoma

**DOI:** 10.1111/jsap.13837

**Published:** 2025-03-23

**Authors:** S. Jeon, G. Lee, N. Lee, D. Chang

**Affiliations:** ^1^ College of Veterinary Medicine Chungbuk National University Cheongju Republic of Korea; ^2^ Haemaru Referral Animal Hospital Seongnam Republic of Korea

## Abstract

Two dogs with haematuria and frequent urination were referred to our veterinary hospital. They were diagnosed with lower urinary tract carcinoma based on urine cytology and BRAF mutation testing. Transarterial embolisation was performed because of persistent haematuria. This procedure involved super‐selective catheterisation and embolisation of the tumour‐feeding arteries using gelatine sponge particles, achieving near stasis. After transarterial embolisation, both patients showed resolution of haematuria within 4 days and a marked reduction in tumour volume after 1 month. However, both patients experienced recurrence of haematuria 4 to 6 weeks after the procedure, leading to a second embolisation being performed for each. Following the second embolisation, the haematuria resolved again. Transarterial embolisation could provide benefits for managing persistent haematuria and provides local tumour control in dogs with lower urinary tract carcinoma.

## INTRODUCTION

Malignant neoplasia of the lower urinary tract in dogs is an uncommon disease with a poor prognosis, most commonly originating from the bladder and urethra (urothelial carcinoma) or the prostate (adenocarcinoma) (Axiak & Bigio, 2012; Mutsaers et al., 2003; Norris et al., 1992). Clinical signs associated with lower urinary carcinomas include haematuria, incontinence, dysuria and nonspecific symptoms such as anorexia and weight loss. Urethral obstruction can result from urethral, bladder or prostate neoplasia, leading to urine retention (Axiak & Bigio, 2012). Traditional treatments of lower urinary tract neoplasia include surgery, nonsteroidal anti‐inflammatory drugs (NSAIDs), chemotherapy, radiotherapy or a combination of these modalities (Fulkerson & Knapp, 2019). Recently, intra‐arterial administration of chemotherapy has emerged as an alternative to traditional therapy for canine lower urinary carcinomas, demonstrating superior tumour response compared to intravenous (iv) chemotherapy (Culp et al., 2015). Additionally, prostatic artery embolisation has been reported to reduce urinary clinical symptoms, including haematuria and decreased tumour size (Culp et al., 2021).

Persistent haematuria from the lower urinary tract, also named “intractable haematuria,” may pose a life‐threatening risk and can arise from various factors, including coagulopathies, neoplasia, inflammation and trauma (Forrester, 2004; Weisse & Berent, 2015). Historically, invasive approaches such as surgical debulking or vessel ligation have been employed to treat human patients with bladder haemorrhage (Niekamp et al., 2017). Persistent haematuria caused by lower urinary tract malignancy in humans, which is not amenable to surgery or involves other comorbidities contraindicating anaesthesia, poses an important life‐threatening problem. In patients with severe bleeding and symptomatic anaemia, continuous transfusions are necessary (Abt et al., 2013). Although several treatment options exist, such as the medical management of acute bleeding, bladder irrigation with saline, alum, formalin or bladder hydrodistension, these methods are not always effective and surgical procedures may be associated with a high risk of morbidity (Choong et al., 2000; Hekimoglu et al., 2023). Owing to the risks associated with these invasive techniques, minimally invasive procedures such as transarterial embolisation (TAE), cystoscopic ablation and radiation therapy have emerged as valuable therapeutic options (Pereira & Phan, 2004). TAE was first described in 1974 for managing intractable refractory haemorrhages when other treatments were not viable (Chen et al., 2021; Hald & Mygind, 1974; Korkmaz et al., 2016; Niekamp et al., 2017).

In veterinary medicine, TAE has been reported as effective in controlling haemorrhage in cases of persistent epistaxis, hepatic tumours, prostatic carcinomas and gluteal pouch haemorrhage (Culp et al., 2021; Delfs et al., 2009; Kawamura et al., 2021; Leveille et al., 2000; Weisse et al., 2004). Additionally, a few human preclinical studies have explored the use of TAE in canine hormone‐induced prostatic hyperplasia models, demonstrating significant prostate shrinkage in some dogs without serious complications (Jeon et al., 2009; Sun et al., 2011). Although previous results do exist for the use of TAE in canine prostatic carcinoma, there were no reports describing it in canine bladder carcinoma. Therefore, this report aims to describe the application of TAE in managing intractable haematuria and control tumour size in dogs diagnosed with lower urinary tract carcinoma.

## CASE HISTORIES

We present two cases of dogs with lower urinary tract carcinoma treated with TAE for persistent haematuria. We conducted a comprehensive search of medical records from 2020 to 2023. Consent for performing TAE was obtained from the owner, and written consent from the dog's owner was obtained for medical data to be used for educational and research publication purposes.

### Case 1

**FIG 1:**
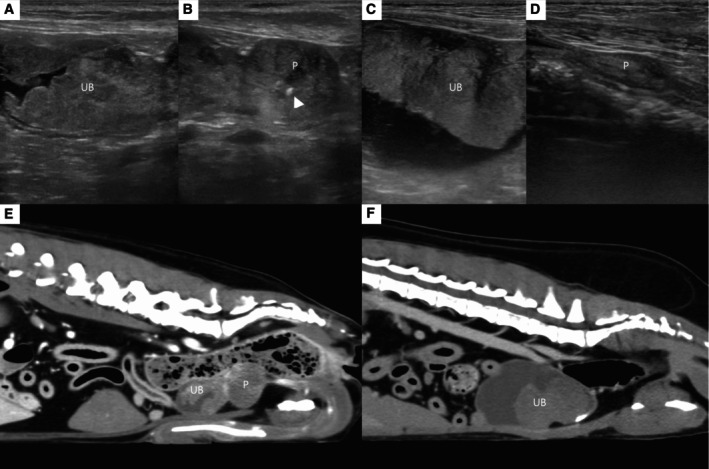
Pre‐treatment ultrasonography (A to D) and CT images (E, F): (A, B) In case 1, a heterogeneous bladder (UB) mass with an irregular contour was observed, and the prostate (P) showed enlargement with focal mineralisation (arrow head). (C, D) In case 2, an iso‐ to hyperechoic mass was observed on the ventral bladder wall, and the prostate appeared normal. (E) In the sagittal CT image of case 1, both masses appeared contiguous. (F) In case 2, the bladder mass was observed on ventral wall.

A 9‐year‐old neutered male toy poodle, weighing 6.3 kg, was referred to our veterinary hospital because of haematuria. An abdominal ultrasound conducted at the referring hospital revealed a prostatic mass with presumed invasion to the urinary bladder. The dog was referred to our hospital for further evaluation of prostate and urinary bladder masses. The initial complete blood count showed no significant abnormalities. Serum biochemistry indicated a mild increase in alanine aminotransferase (140.36 U/L; reference range, 19 to 70 U/L), with no other abnormalities observed. Abdominal ultrasonography showed heterogeneous hypoechoic enlargement of the prostatic with mineralisation, measuring 13.8 × 20.3 mm in the maximal sagittal plane (Fig. 1). The urinary bladder neck was thickened with irregular margins. The bladder mass was connected to the prostate mass with irregular margins and mineralisation. Urine cytology following traumatic urethral catheterisation revealed large pleomorphic cells with oval nuclei. Based on the cytological and ultrasonographic findings, the prostatic mass was diagnosed as a prostatic carcinoma with invasion of the urinary bladder. CT scan was performed to investigate the characteristics of the urinary bladder and prostate masses and check for potential metastases. The CT scan utilised a 16‐row multi‐detector CT scanner (Somatom Emotion, Siemens Medical Systems, Forchheim, Germany) with a slice thickness of 0.75 mm, applying 110 mAs and 110 kV. A triple‐phase contrast study followed the iv administration of 880 mgL/kg iohexol (Omnipaque 300®, GE Healthcare, Shanghai, China) at 3 mL/s using a power injector (Medrad Vistron C‐T Injector System, Medrad, Inc., Minneapolis, USA). A hypo‐ to iso‐attenuating prostatic mass with mineralisation was observed, similar to the ultrasonography findings; the prostatic mass was continuous with the bladder mass, which also showed hypo‐ to iso‐attenuation. The tumour volume, calculated using a DICOM viewer (OsiriX MD, version 13.0.1, Pixmeo SARL, Switzerland), was 16.94 cm^3^.

### Case 2

**FIG 2:**
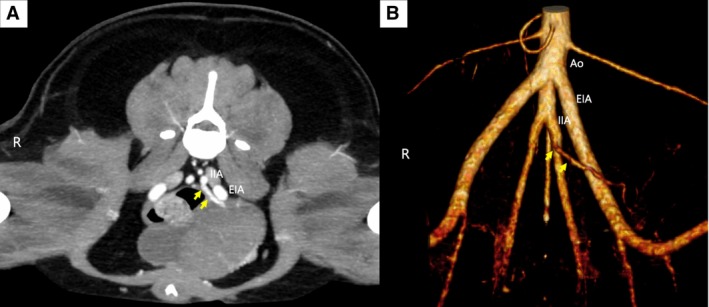
Transverse CT image (A) and volume rendering image (B) of case 2 reveal the presence of an umbilical artery (yellow arrow) originated from the left internal iliac artery (IIA). Ao Aorta, EIA External iliac artery.

An 8‐year‐old neutered male bichon frise weighing 10.5 kg was admitted for further evaluation of a previously diagnosed urinary bladder tumour. At the referring hospital, the dog was examined for chief complaints of frequent urination and haematuria. The dog was referred to our hospital for further evaluation. Upon initial haematological examination, mild to moderate regenerative anaemia was noted (HCT 27.7%; reference range, 37.3% to 61.7%). Additionally, the platelet count was at the lower limit of the normal range (185 k/μL; reference range, 148 to 484 k/μL). Serum biochemistry results indicated mild elevations in alanine aminotransferase levels (99.57 U/L; reference range, 19 to 70 U/L), blood urea nitrogen (33.99 mg/dL; reference range, 8 to 26 mg/dL) and fasting glucose (152 mg/dL; reference range, 70 to 118 mg/dL). Abdominal ultrasonography revealed an iso‐ to hypo‐echoic heterogeneous solid urinary bladder mass with significant vascularisation. Urine cytology revealed clusters of large, polygonal and round cells with high nucleus‐to‐cytoplasm ratios. A canine BRAF mutation test using voided urine yielded positive results. Based on these results, urothelial carcinoma was diagnosed. CT scan revealed a bladder mass in the body without adjacent organ invasion, with a tumour volume of 37.58 cm^3^. An umbilical artery extending from the internal iliac artery was detected, prominently supplying vascularisation to the bladder mass (Fig. 2). The arterial supply to the urinary bladder mass originated from the bilateral caudal vesical and umbilical arteries.

## TAE PROCEDURES AND POSTOPERATIVE PROGNOSIS

**FIG 3:**
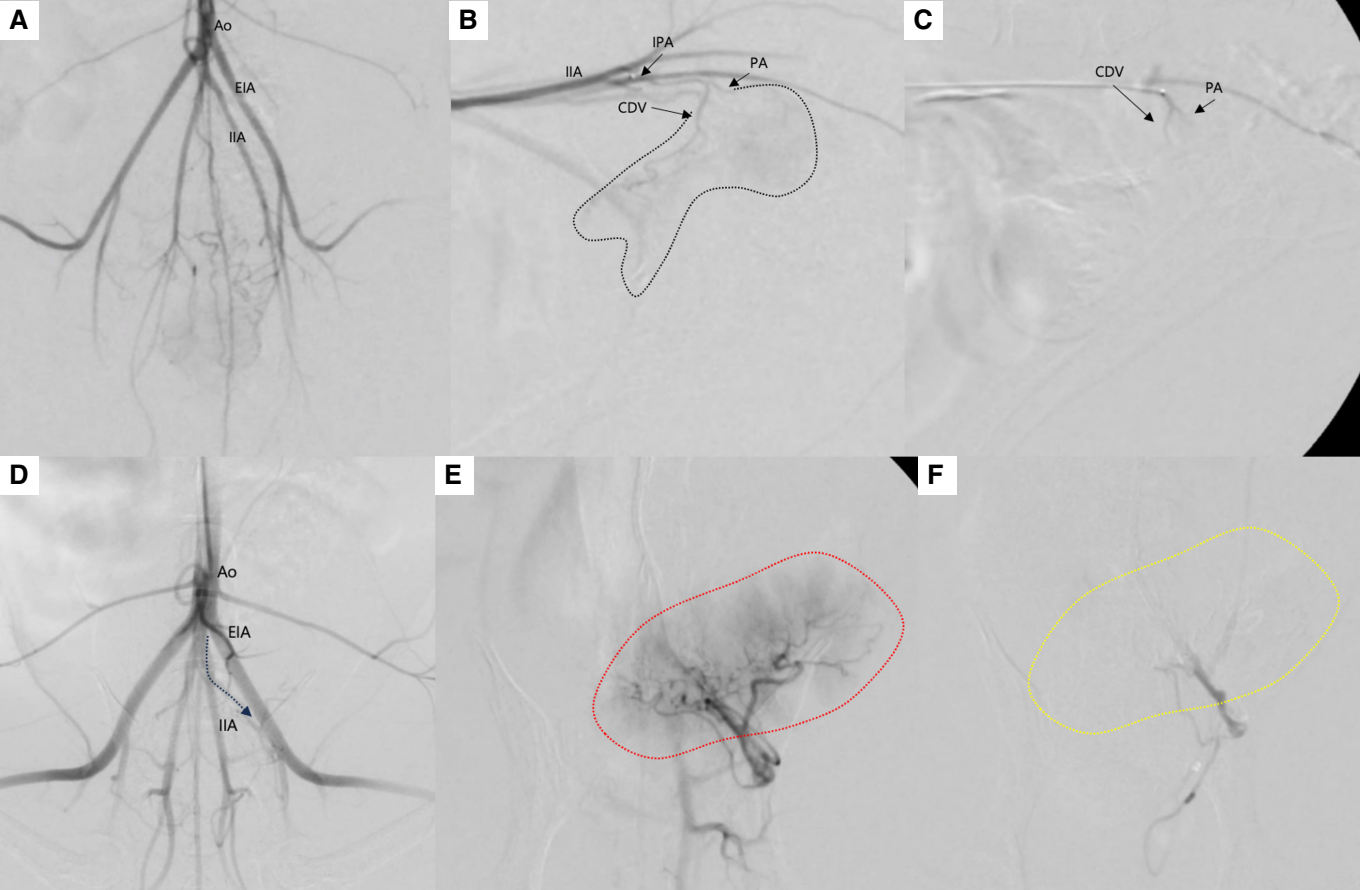
Digital subtraction images: (A to C) In case 1, (A) Distal aorta (Ao) angiogram. (B) Right internal iliac artery (IIA) angiogram shows tumour staining from the prostatic artery (PA) and caudal vesical artery (CDV) (black dotted line). (C) Prostatic artery angiogram after embolisation shows loss of tumour staining. (D to F) In case 2, (D) In aorta angiogram, the umbilical artery (dotted arrow) was seen. (E) Angiogram of the umbilical artery shows tumour staining with small tortuous vessels (red dotted line). (F) Umbilical artery angiogram after embolisation shows tumour staining diminished (yellow dotted line). EIA External iliac artery, IPA Internal pudendal artery.

TAE was performed under general anaesthesia in both dogs. Before anaesthesia, premedication was administered iv: 0.2 mg/kg dexamethasone disodium phosphate (Jeil Dexamethasone, Jeil, Daegu, Korea) and 25 mg/kg cefazolin sodium (Cefazolin, Chongkundang, Seoul, Korea). The equipment used in TAE is summarised in Table 1. A transarterial cut‐down approach via the carotid artery was performed using a 5‐French introducer sheath. A 4‐French‐angled angiographic catheter was introduced using a 0.035‐inch guide wire through the carotid artery and thoracic aorta, reaching the level of the caudal aorta (Fig. 3). The guidewire was withdrawn from the angiographic catheter and under fluoroscopic guidance, 3 mL of contrast agent was injected to visualise the vascular anatomy of the terminal aorta. Superselection of the prostatic and caudal vesical artery was performed using a 0.014‐inch microwire and 1.5‐French microcatheter to access the internal iliac and internal pudendal arteries. The patient was placed in the lateral recumbent position. Subsequently, 1 mL of contrast agent was injected to delineate the prostatic and caudal vesical artery.

**Table 1:** Equipment used in transarterial embolisation

Equipment	Name	Characteristic	Manufacturer	Country
Introducer sheath	Prelude	5‐French, 4 cm	Merit Medical	South Jordan, USA
Angiographic catheter	KMP	4‐French angled, 65 cm	Jungsung Medical	Seoul, Korea
Guide wire	Zip wire	0.035‐in., 150 cm	Boston Scientific	Marlborough, USA
Microwire	Meister S14	0.014‐in., 135 cm	Asahi Intecc	Nagoya, Japan
Microcatheter	Veloute ultra	1.5‐French, 105 cm	Asahi Intecc	Nagoya, Japan
Gelatine sponge particles	Marin‐Gel S	100 to 150 μm	PL Micromed	Yangsan, Korea
Fluoroscopy	OEC Elite CFD		GE Healthcare	Chicago, USA

In case 1, the right and left prostatic and caudal vesical arteries were embolised using 100 to 150 μm gelatine sponge particles (GSPs) dissolved in 10 mL of contrast agent. The selected vessels were embolised to achieve near stasis. An internal iliac angiogram was obtained after embolisation to ensure blockage of the targeted vessels. The total volume of infused embolic agent solution was 0.4 mL in the right prostatic and caudal vesical arteries and 0.6 mL in left prostatic and caudal vesical arteries. In case 2, because of the arterial supply from the umbilical artery, an embolisation procedure for the bilateral caudal vesical arteries and umbilical artery was performed, as in case 1. The total volume of infused embolic agent was 0.4 mL in the right caudal vesical artery, 0.5 mL in left caudal vesical artery and 2.0 mL in umbilical artery. Following the removal of the microcatheter and angiographic catheter, the introducer sheath was withdrawn. The carotid artery was ligated, and the skin was closed using routine techniques. Both dogs recovered from the anaesthesia without any complications. Both dogs were closely monitored after the procedure, and no adverse events, such as pain or haemorrhage, were observed. To manage post‐procedural discomfort and prevent potential complications such as post‐embolisation syndrome, we prescribed the following medications: 2 mg/kg maropitant once daily (Cerenia, Zoetis, Kalamazoo, USA), 10 mg/kg gabapentin twice daily (Neurontin, Viatris, Seoul, Korea) and 12.5 mg/kg amoxicillin‐clavulanic acid twice daily (Amocla, Kuhnil, Seoul, Korea) for 1 week.

**FIG 4:**
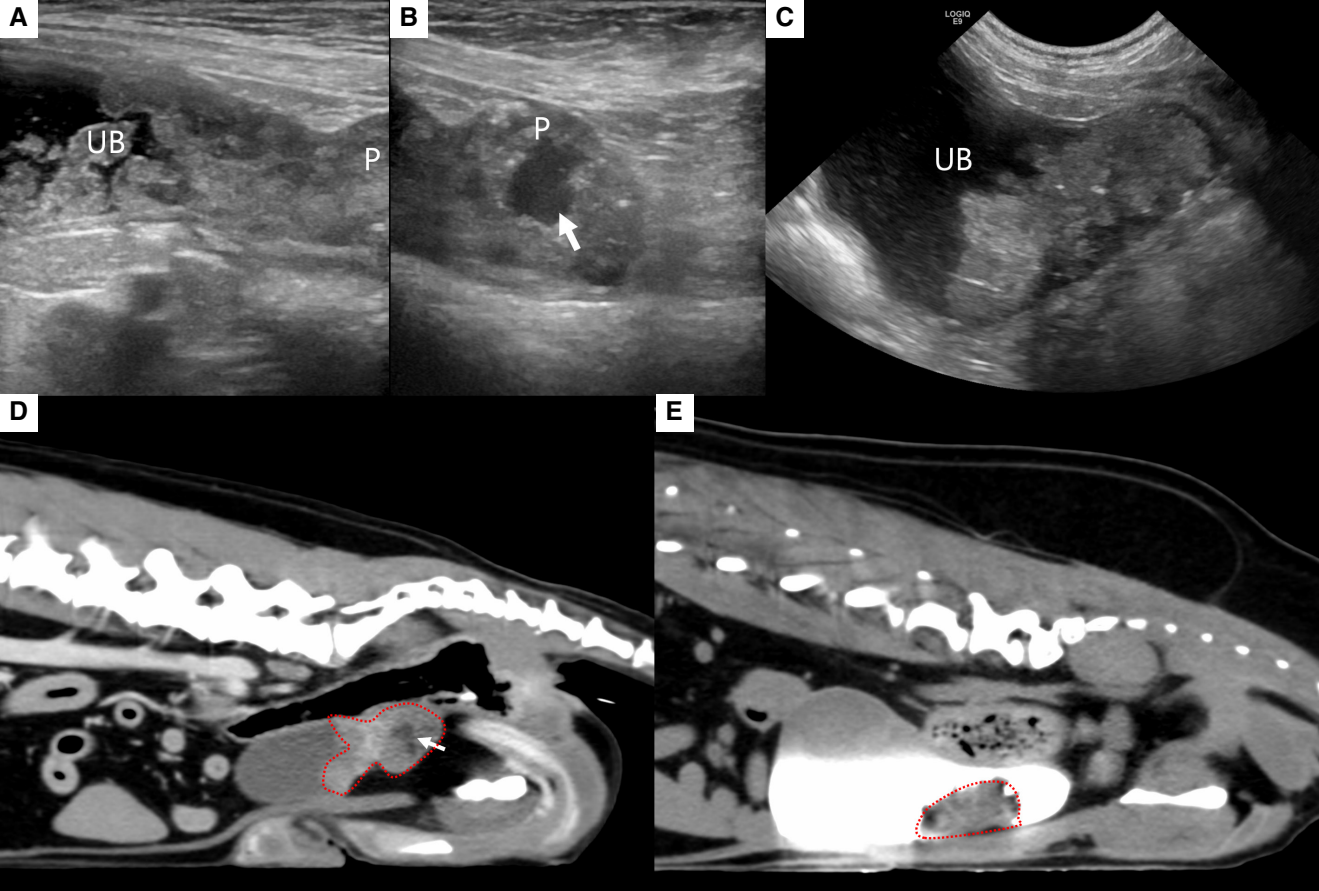
Post‐treatment ultrasonography (A to C) and CT images (D, E): (A, B, D) In case 1, the bladder (UB) and prostatic (P) masses were decreased in size. Focal necrotic area was observed in prostate (arrows) in both images. (C, E) In case 2, the bladder mass showed a marked decrease in size.

Both dogs showed significant improvement in symptoms, including haematuria and pollakiuria, within 3 days (case 1) and 4 days (case 2) after the TAE procedure. Postoperative CT performed 1 month after the initial TAE revealed a decrease in tumour volume in both cases (Fig. 4). Case 1 showed a 58% tumour reduction to 7.03 cm^3^, and Case 2 had a 60.9% reduction to 14.67 cm^3^. Additionally, in case 2, the anaemia resolved, and platelet levels normalised (334 k/μL).

**FIG 5:**
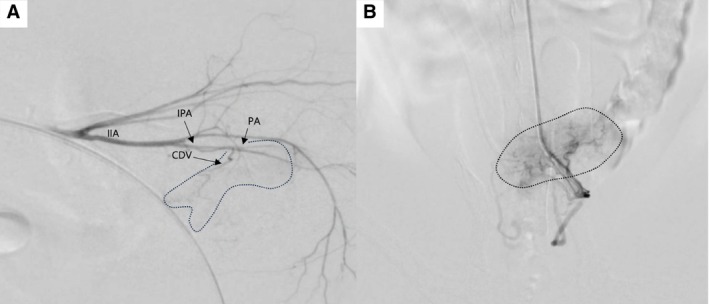
Digital subtraction angiographic images obtained during second TAE procedure in case 1 (A) and case 2 (B). (A) Selective angiography of the right internal iliac artery. (B) Selective angiography of the left umbilical artery. Both images shows that the small tortuous vessels supplying the tumour had decreased compared to the first procedure, and decreased size in tumour staining. CDV Caudal vesical artery, IIA Internal iliac artery, IPA, Internal pudendal artery; PA Prostatic artery.

In Case 1, following the TAE procedure, iv chemotherapy was initiated with carboplatin at intervals of 3 to 4 weeks. Additionally, the dog received daily oral doses of 0.3 mg/kg piroxicam (Piroxicam Cap.; GL Pharma, Seongnam, Korea). Approximately 6 weeks later, haematuria recurrence was observed. Consequently, a repeated TAE procedure was performed via the left femoral artery using the same method. As with the first procedure, the selected vessels were embolised to achieve near stasis. On fluoroscopy during the second procedure, the small tortuous vessels supplying the tumour had decreased compared to the first procedure (Fig. 5). After the second TAE, haematuria resolved, indicating a positive response to treatment. However, 193 days after the first TAE, owing to the presence of pulmonary metastases and a significant decline in the dog's quality of life, the owner chose to euthanise the dog to prevent further suffering. There was no recurrence of haematuria until the time of euthanasia. In case 2, the owner refused systemic chemotherapy and oral NSAIDs due to potential side effects of the systemic medical therapy. Haematuria recurred 4 weeks after the initial TAE, prompting a repeated TAE procedure via the right carotid artery using the same technique. Similar to case 1, the small tortuous vessels supplying the tumour had decreased compared to the previous procedure, and due to the tumour size reduction, the tumour staining on the selective angiogram of the aberrant artery also diminished. After the second procedure, the haematuria disappeared again, with no signs of urinary retention. However, mild intermittent haematuria reappeared in the 7th week following the second TAE. Despite this, the patient's overall vitality remained stable, and it was still alive 352 days after the first TAE procedure, at the time of manuscript.

## DISCUSSION

This study investigated using TAE to treat lower urinary tract carcinoma in dogs, aiming to manage haematuria and reduce tumour size. Some human studies have shown that TAEs can improve haematuria in patients with severe bladder haemorrhage in malignancies (Chen et al., 2021; De Berardinis et al., 2005; Hald & Mygind, 1974; Loffroy et al., 2014; Pereira & Phan, 2004). Most studies on endovascular embolisation in humans for severe haematuria are small case series, with a high technical success rate ranging from 92.6% to 100% (Chen et al., 2021). In this study, no technical difficulties were encountered in either dog and clinical improvement was observed within 4 days of the procedure.

In humans, TAE is a key treatment for non‐resectable tumours, especially hepatocellular carcinoma (Rammohan et al., 2012). The principal concept involves the intentional endovascular occlusion of the feeding arteries of the tumour, which leads to necrosis, ischaemia and infarction of the tumour tissue, thereby reducing its size and burden (Guimaraes et al., 2014). Similarly, embolisation resolved bladder haematuria and reduced the tumour size of the two patients in this study. In case 2, an aberrant vessel originating from the umbilical artery was identified as a significant contributor to bleeding on triple‐phase CT images and selective angiogram on fluoroscopy images. During angiography, the procedure revealed tumour‐feeding vessels from the both caudal vesical arteries, prompting the decision to embolise both the aberrant and caudal vesical arteries.

In the presented cases, the embolisation endpoint was set to near stasis rather than complete stasis to avoid ischaemic damage to the bladder wall. In a human TAE study, near stasis refers to a reduced blood flow, where the contrast medium takes a considerably longer time to clear from the embolised vessel than from an adjacent non‐embolised vessel (*i.e*. for the contrast column to clear within 2 to 5 heartbeats on completion arteriography) (Lee et al., 2017). Although the effectiveness and durability of embolization may be less than those of complete embolisation, it can relatively reduce ischaemic complications such as bladder necrosis or non‐target embolisation. Although the parent vessels were embolised with near stasis, angiogram images from the second procedure in each patient showed that the small tortuous vessels supplying the tumour had disappeared and tumour staining had decreased as a result of the tumour size reduction.

In veterinary medicine, TAE and transarterial chemoembolisation have been used for the treatment of persistent haemorrhage and as palliative therapy for unresectable tumours (Cave et al., 2003; Culp et al., 2021, 2022; Kawamura et al., 2021; Kimata et al., 2022; Marioni‐Henry et al., 2007; Rogatko et al., 2021; Weisse et al., 2002, 2004). Various embolic materials have been used, including GSPs, microspheres and polyvinyl alcohol. However, consensus on the optimal material for embolisation in veterinary medicine remains lacking. GSPs cause mechanical obstruction, slow blood flow and accelerate thrombus formation. GSPs embolisation results in temporary vessel occlusion, with recanalisation occurring within a few weeks (Vaidya et al., 2008). This temporary occlusion can be advantageous for haemorrhagic embolisation, and the procedure can be repeated because of recanalisation. In humans, non‐target embolisation at other sites of the internal iliac territory with permanent embolic agent such as microspheres can make bladder ischemia and necrosis, which are possible serious complications (Delgal et al., 2010; Tarkhanov et al., 2018). The retrospective study of prostatic artery embolisation using microsphere in dogs with prostatic carcinoma, was also limited to embolising only the prostatic artery, even in cases where the bladder was invaded, to prevent bladder necrosis (Culp et al., 2021). To address this risk, we used GSPs, a resorbable embolic material to achieve near stasis embolisation and reduce side effects. This approach not only effectively managed persistent bleeding but also minimised the risk of bladder ischaemia. This approach utilising near stasis and resorbable embolic materials offers advantages, such as minimising side effects and allowing revascularisation of the parent artery, which permits additional procedures if needed. However, it has limitations, including shorter haemostasis duration and less effective tumour response. Both patients experienced recurrence of haematuria at 4 and 6 weeks, respectively, and consequently underwent a second TAE procedure. Achieving a more favourable tumour response may require a more aggressive embolisation technique, such as complete stasis embolisation or the use of a permanent embolic agent. However, in this case series, we opted for a safer approach aimed at minimising complications such as bladder ischaemia, even if it necessitated additional embolisation procedures.

Embolisation not only resolved bladder haematuria but also reduced the tumour size of the two patients in this study. Although the primary aim of TAE was to control bleeding, the procedure showed promising short‐term antitumor responses and local control. NSAIDs are commonly used to treat urogenital carcinomas. In dogs with prostatic carcinoma, NSAID treatment alone has been associated with a median overall survival time (mOST) of 6.9 months (Sorenmo et al., 2004). Combining chemotherapy with NSAID treatment is also a common practice and has been shown to improve mOST in dogs with prostatic carcinoma to 106 days and in those with bladder carcinomas to 291 days (Henry et al., 2003; Ravicini et al., 2018). Surgical intervention can alleviate clinical signs related to local disease and often results in longer mOST compared to medical treatments (Bennett et al., 2018; Marvel et al., 2017). However, many lower urinary tract tumours involve the trigone of the bladder or the ureters, necessitating complete cystectomy with urinary diversion. Although this surgical approach is feasible, it is frequently declined by owners due to the potential morbidity associated with the procedure and the impact on the patient's quality of life. The use of palliative stents in urethral obstruction due to malignancy is available (Blackburn et al., 2013; McMillan et al., 2012). Median survival times (MSTs) extended to 251 days when treatment involved NSAIDs prior to or chemotherapy after urethral stent placement for malignant obstruction, in contrast to cases without medical therapy (Blackburn et al., 2013). Ureteral stenting is also an option to relieve malignant obstruction when a lower urinary tract carcinoma arising from the bladder, urethra or prostate invades the ureteral opening within the bladder (Berent et al., 2011). Recent studies have shown that intensity‐modulated and image‐guided radiation therapy can provide clinical benefits in 90% of dogs with urogenital carcinomas, achieving a MST of 654 days (Nolan et al., 2012). Another study involving 51 dogs with urogenital carcinoma treated with radiation therapy, with or without additional medical treatment, reported a mOST of 510 days (Clerc‐Renaud et al., 2021). However, radiation therapy also has limitations, including high costs, the risks associated with repeated anaesthetic procedures, and potential acute and late toxicities such as diarrhoea and ureteral or urethral strictures (Clerc‐Renaud et al., 2021; Nolan et al., 2012). In case 1, the dog had an advanced stage of the disease, achieved a survival time of 193 days with a combination of TAE and chemotherapy, and the cause of death was attributed to pulmonary metastasis rather than local progression. Although the survival time was not superior compared to the MST observed with NSAID monotherapy, there was no recurrence of haematuria after the second procedure until the time of death. This outcome is notable for a late‐stage disease, especially considering that palliative care for symptoms like haematuria can be beneficial. In case 2, despite not receiving any additional medical treatment, the dog has survived 352 days with repeated TAE alone. Although mild haematuria recurred after the second TAE procedure, there was no severe haematuria that could have resulted in anaemia or thrombocytopenia during the remaining survival period. This suggests that the local tumour control ability of TAE played a significant role, resulting in a longer survival time compared to the MST with medical treatment.

Transarterial embolisation is beneficial for managing persistent haematuria associated with lower urinary tract carcinoma and may have potential for controlling tumour size in the presented patients. Although haematuria may recur, it can be resolved through repeated TAE procedures. Further studies are needed to evaluate long‐term tumour volume monitoring and survival benefits.

### Author contributions

**S. Jeon:** Conceptualization (lead); data curation (lead); investigation (lead); methodology (lead); validation (lead); writing – original draft (lead). **G. Lee:** Conceptualization (supporting); investigation (supporting); supervision (supporting); writing – original draft (supporting). **N. Lee:** Data curation (supporting); investigation (supporting); supervision (supporting); writing – review and editing (supporting). **D. Chang:** Conceptualization (supporting); data curation (supporting); supervision (lead); validation (supporting); writing – review and editing (lead).

### Conflict of interest

None of the authors of this article has a financial or personal relationship with other people or organisations that could inappropriately influence or bias the content of the paper.

## Data Availability

The data that support the findings of this study are available on request from the corresponding author. The data are not publicly available due to privacy or ethical restrictions.
